# A comparison of sex steroid concentration levels in the vitreous and serum of patients with vitreoretinal diseases

**DOI:** 10.1371/journal.pone.0180933

**Published:** 2017-07-13

**Authors:** Yuko Nishikawa, Seita Morishita, Taeko Horie, Masanori Fukumoto, Takaki Sato, Teruyo Kida, Hidehiro Oku, Jun Sugasawa, Tsunehiko Ikeda, Kimitoshi Nakamura

**Affiliations:** 1 Department of Ophthalmology, Osaka Medical College, Takatsuki-City, Osaka, Japan; 2 Nakamura Eye Clinic, Matsumoto-City, Nagano, Japan; Bascom Palmer Eye Institute, UNITED STATES

## Abstract

The purpose of this study was to compare steroid hormone concentration levels in the vitreous and serum of vitreoretinal disease patients to elucidate the possibility of neurosteroid production in the retina. Serum and vitreous samples were collected from vitrectomy patients, and estradiol (E2) and testosterone (T) concentrations were measured using electro-chemiluminescence immunoassay. We measured E2 in epiretinal membrane (ERM, n = 14), macular hole (MH, n = 18), proliferative diabetic retinopathy (PDR, n = 20), and retinal detachment (RD, n = 19) cases, and T in ERM (n = 14), MH (n = 17), PDR (n = 13), and RD (n = 17) cases. No statistically significant age differences existed among the groups. Mean respective E2 concentrations (pg/ml) in the male/female vitreous were ERM: 6.67±4.04/18.82±7.10, MH: 10.3±7.02/17.00±4.8, PDR: 4.2±3.05/15.83±3.46, and RD: 10.00±4.58/16.06±4.57, while those in serum were ERM: 31.67±5.51/5.82±1.08, MH: 21.00±8.89/7.53±3.2, PDR: 29.20±7.07/12.75±10.62, and RD: 24.33±6.51/7.5±4.42. E2 concentrations were significantly higher (*P*<0.001) in the male serum than vitreous, yet significantly higher in the female vitreous than serum. Mean respective T concentrations (ng/ml) in the male/female vitreous were ERM: 0.15±0.03/0.15±0.01, MH: 0.15±0.01/0.15±0.01, PDR: 0.15±0.03/0.16±0.12, and RD: 0.14±0.01/0.17±0.08, while those in serum were ERM: 4.54±1.46/0.16±0.01, MH: 8.04±2.29/0.16±0.10, PDR: 5.14±1.54/0.22±0.11, and RD: 3.24±0.75/0.17±0.10. T concentrations were high in the male serum, yet extremely low in the male and female vitreous and female serum. High concentrations of E2 were found in the vitreous, and women, in particular, exhibited significantly higher concentrations in the vitreous than in the serum. This finding suggests the possibility that in vitreoretinal disease cases, the synthesis of E2 is increased locally only in female eyes.

## Introduction

The brain has traditionally been perceived as the target organ of steroid hormones synthesized by the peripheral endocrine organs, such as the adrenal glands and reproductive organs. However, Baulieu et al [1] and Tsutsui et al [2,3] reported that the brain also produces steroid hormones on its own. An enzyme (P450scc) that synthesizes cholesterol to pregnenolone (P5) in the retina is present in retinal ganglion cells (RGCs) and is reportedly involved in the production of neurosteroids [4]. It has also been reported that estrogen and androgen affect retinal thickness, raising the possibility that sex steroids play an important role in the retina [4,5].

Estradiol (E2) is converted from testosterone (T) and androstenedione by aromatase, and there have been reports of aromatase manifesting in retinal pigment epithelial cells and sensory retina [5,6]. Moreover, E2 is thought to have neuroprotective effects [7–10]. In this present study, we measured and compared the concentration levels of steroid hormones E2 and T in the serum and vitreous body of patients afflicted with different types of vitreoretinal diseases that were subject to vitrectomies in order to explore the possibility of sex steroid hormone production in the eye.

## Materials and methods

In this study, the following four vitreoretinal diseases were examined: idiopathic epiretinal membrane (ERM), idiopathic macular hole (MH), proliferative diabetic retinopathy (PDR), and rhegmatogenous retinal detachment (RD). Measurements of E2 were taken in a total of 71 patients seen at the Department of Ophthalmology, Osaka Medical College Hospital, Takatsuki City, Japan from April 2013 through August 2016: 14 ERM patients (3 males and 11 females), 18 MH patients (3 males and 15 females), 20 PDR patients (10 males and 10 females), and 19 RD patients (3 males and 16 females). Measurements of T were taken in a total of 61 patients seen at the Department of Ophthalmology, Osaka Medical College Hospital from April 2013 through August 2016: 14 ERM patients (12 males and 2 females), 17 MH patients (2 males and 15 females), 13 PDR patients (9 males and 4 females), and 17 RD patients (6 males and 11 females). Since sample volumes were insufficient, we were unable to measure both E2 and T in each patient. The mean age (± standard deviation) of the male and female E2-measurement patients was 61.9 ± 14.8 years (range: 53–76 years) and 67.4 ± 6.7 years (range: 56–86 years), respectively, while that of the male and female T-measurement patients was 58.6 ± 15.5 years (range: 48–71 years) and 64.5 ± 10.6 years (range: 52–79 years), respectively. There was no statistically significant age difference among the groups. All operations were performed at Osaka Medical College Hospital. This study was approved by the Ethics Committee of Osaka Medical College, and was performed in accordance with the tenets set forth in the Declaration of Helsinki. Informed written consent was obtained from all subjects prior to the preoperative blood test examination and the vitrectomy being performed.

Vitreous fluid (0.6–1.0ml) was collected into sterile tubes during vitreous surgery without contamination by balanced saline solution, and was rapidly frozen to -80°C. Electro-chemiluminescence immunoassay (ECLIA; SRL, Inc., Tokyo, Japan) was used to measure the concentrations of E2 and T. Measurements were made by first adding biotinylated antibodies, E2 antibodies, and T antibodies to the specimen to induce a reaction, followed by adding streptavidin-coated magnetic microparticles to create a reaction. The reaction mixture was then absorbed into the cell being measured. In addition, the emission intensities of E2 and T bound to the streptavidin-coated magnetic microparticles were measured using a photomultiplier tube. The concentrations of E2 and T in the sample specimen were extrapolated from the emission intensity of a calibrator operating in the same manner.

The data were expressed by mean ± standard deviation (SD). Significant differences between the groups were calculated by paired t-test. The differences were considered significant at *P* < 0.05.

## Results

**Fig 1:**
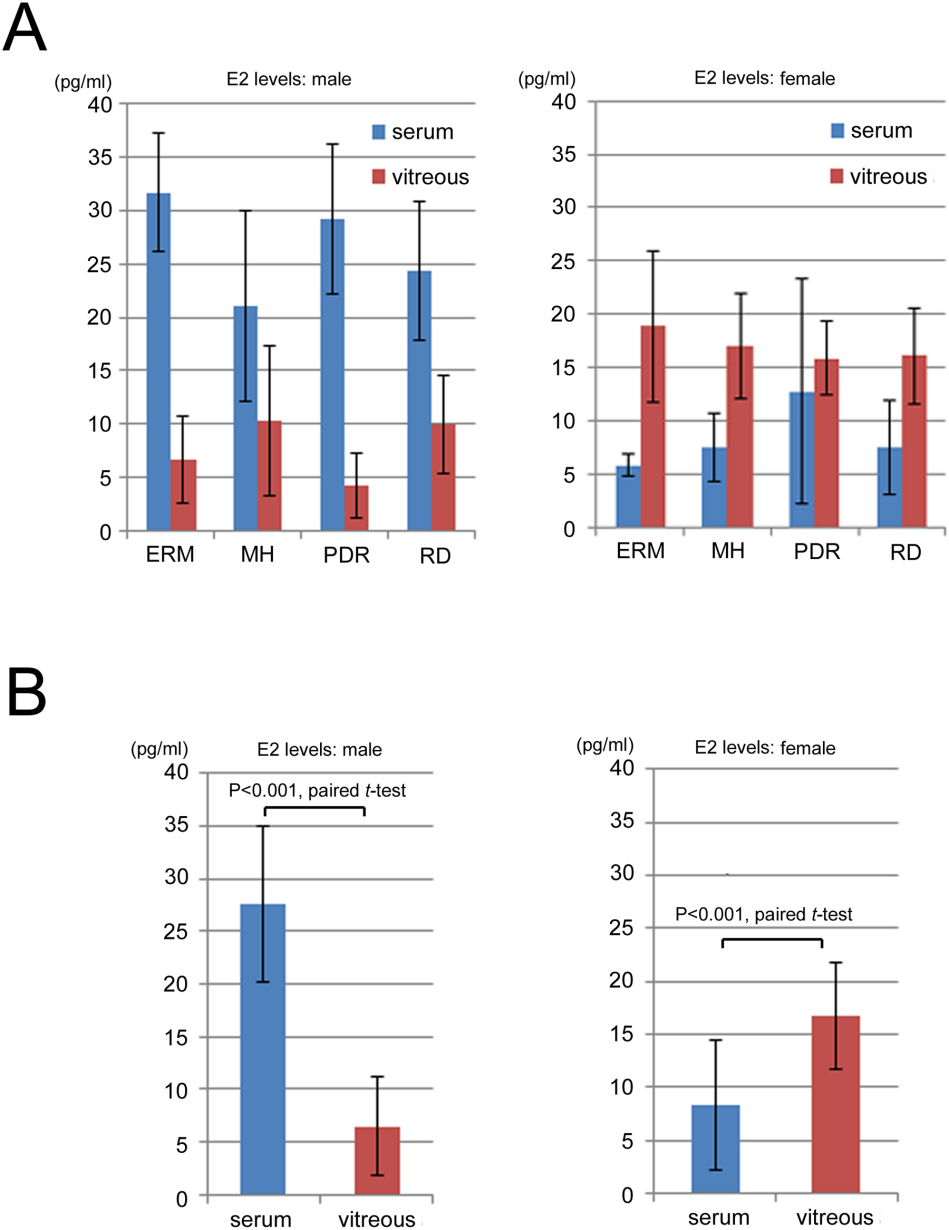
Estradiol (E2) concentrations (pg/ml) in the vitreous body and serum between the male and female patients in the four vitreoretinal disease groups (A), and between the male and female patients (B). In all 4 vitreoretinal diseases, E2 concentrations were significantly higher (*P < 0.001) in the serum than in the vitreous body in the males, while the females exhibited significantly higher E2 concentrations in the vitreous body than in the serum. ERM: idiopathic epiretinal membrane; MH: idiopathic macular hole; PDR: proliferative diabetic retinopathy; RD: rhegmatogenous retinal detachment (RD) (blue: serum, red: vitreous body).

The mean E2 concentrations (pg/ml) in the male vitreous body samples were ERM: 6.67 ± 4.04, MH: 10.33 ± 7.02, PDR: 4.2 ± 3.05, and RD: 10.00 ± 4.58, while those for the female samples were ERM: 18.82 ± 7.10, MH: 17.00 ± 4.8, PDR: 15.83 ± 3.46, and RD: 16.06 ± 4.57. The mean E2 concentrations in the male serum samples were ERM: 31.67 ± 5.51, MH: 21.00 ± 8.89, PDR: 29.20 ± 7.07, and RD: 24.33 ± 6.51, while those in the female samples were ERM: 5.82 ± 1.08, MH: 7.53 ± 3.2, PDR: 12.75 ± 10.62, and RD: 7.5 ± 4.42. The mean E2 concentration for the males across the four diseases was 6.47 ± 5.72 in the vitreous body and 27.53 ± 7.47 in the serum, while the mean E2 concentration for the females across the four diseases was 16.83 ± 5.37 in the vitreous body and 8.33 ± 6.62 in the serum. In all vitreoretinal diseases, the E2 concentration was significantly higher (*P* < 0.001) in the serum than in the vitreous body for the males, while conversely, the females exhibited a significantly higher E2 concentration in the vitreous body than in the serum (Fig 1).

**Fig 2:**
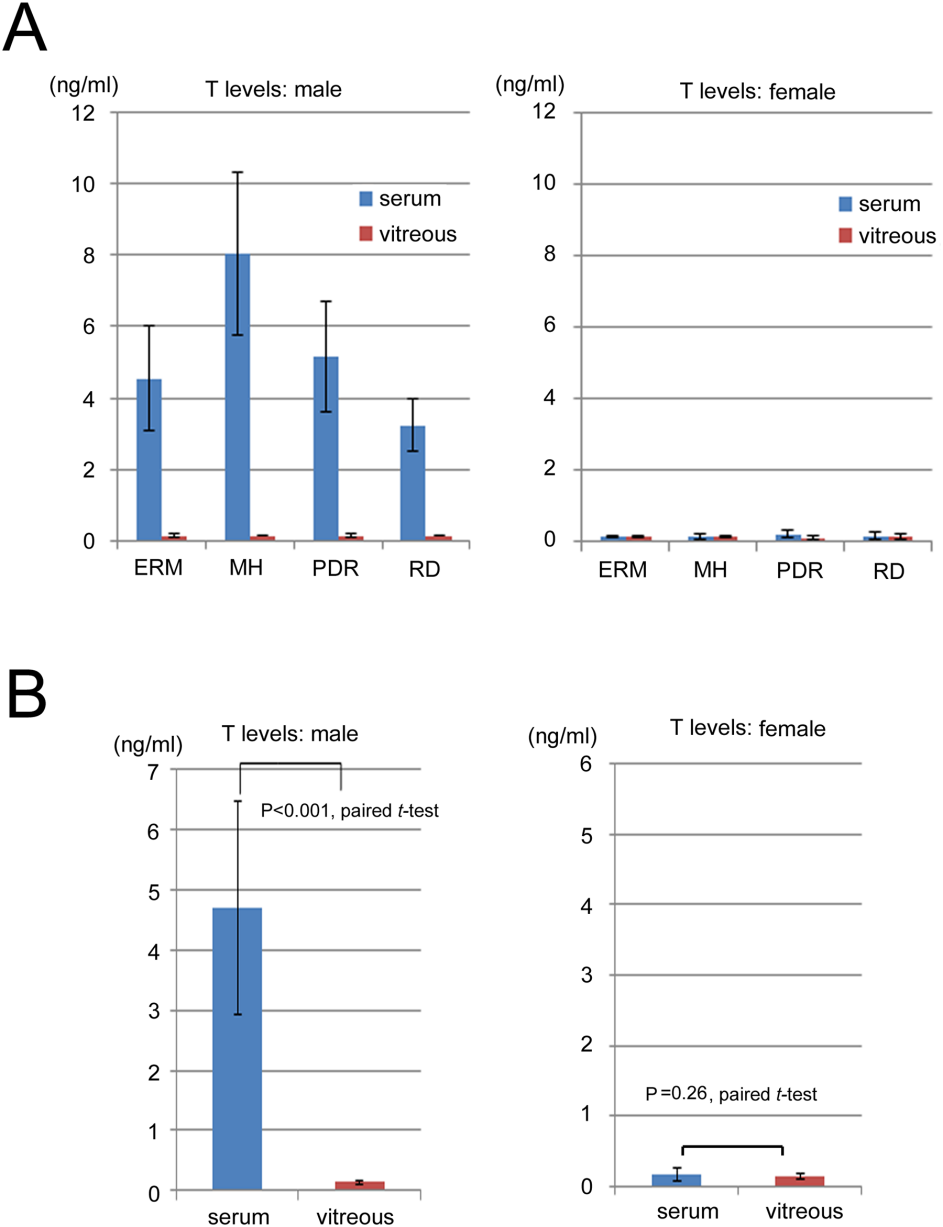
Testosterone (T) concentrations (ng/ml) in the vitreous body and serum between the male and female patients in 4 vitreoretinal disease groups (A) and between the male and female patients (B). T concentration in the serum was significantly higher than that of the vitreous for the males (*P < 0.001). T concentration was extremely low in the vitreous body of the males as well as in the vitreous body and serum of the females. ERM: idiopathic epiretinal membrane; MH: idiopathic macular hole; PDR: proliferative diabetic retinopathy; RD: rhegmatogenous retinal detachment (RD) (blue: serum, red: vitreous body).

The mean T concentrations (ng/ml) in the male vitreous body samples were ERM: 0.15 ± 0.03, MH: 0.15 ± 0.01, PDR: 0.15 ± 0.03, and RD: 0.14 ± 0.01, while those in the female samples were ERM: 0.15 ± 0.01, MH: 0.15 ± 0.01, PDR: 0.16 ± 0.12, and RD: 0.17 ± 0.08. The mean T concentrations (ng/ml) in the male serum samples were ERM: 4.54 ± 1.46, MH: 8.04 ± 2.29, PDR: 5.14 ± 1.54, and RD: 3.24 ± 0.75, while those in the female samples were ERM: 0.16 ± 0.01, MH: 0.16 ± 0.10, PDR: 0.22 ± 0.11, and RD: 0.17 ± 0.10. The mean T concentration for the males across the four diseases was 0.15 ± 0.02 in the vitreous body and 4.70 ± 1.69 in the serum, while the mean T concentration for the females across the four diseases was 0.15 ± 0.05 in the vitreous body and 0.17 ± 0.08 in the serum. Although the mean T concentration in the serum was significantly higher than that of the vitreous for the males (*P* < 0.001), the T concentration was extremely low in the vitreous body of the males as well as in the vitreous body and serum of the females (Fig 2).

## Discussion

It has conventionally been thought that sex steroid hormones produced in the gonads, such as E2 and T, as well as glucocorticoid produced in the adrenal cortex, are not synthesized in the brain but are rather transported to the brain, and in particular the hypothalamus, by the blood, producing an effect on nerve cells (hypothalamic pituitary adrenal/gonadal axis) [11]. In other words, it was believed that the brain was simply the target organ of steroid hormones produced from what began as cholesterol in the peripheral gonads and adrenal glands.

Baulieu et al [1] reported that in adult rats, an independent synthesis system existed in the brain due to the presence of high concentrations of dehydroepiandrosterone (DHEA), a steroid hormone. In addition, they found that the concentrations of DHEA in the brain did not decrease even after extracting the peripheral steroid synthesis organs, such as the adrenal cortex and reproductive organs. Following the findings of Baulieu’s group, there have been reports [12,13] that steroids such as P5 and progesterone existed in higher concentrations in the brain than in the blood, and these have now become known as neurosteroids.

It is thought that neurosteroids are synthesized in the brain and serve different kinds of physiological functions by operating in contained regions. Neurosteroids are particularly known to manifest significant effects in the limbic system, which is deeply tied to memory and emotion, such as in the hippocampus and the amygdala. Kawato et al [14] reported that neurosteroids such as DHEA and P5 sulfate are synthesized in the brain’s hippocampus. Believing that there must be a local synthesis system within the brain if neurosteroids were involved with memory and learning, Kimoto et al [15] further reported an enzyme system in nerve cell layers that synthesizes neurosteroids, such as cytochrome P450scc in the nerve cell layers of the hippocampus, cytochrome P45017α, and cytochrome P450arom (aromatase).

The findings of subsequent studies revealed that numerous steroids are synthesized in the brains of vertebrates, including hydroxysteroid sulfotransferase, 3β-hydroxysteroid dehydrogenase/Δ5–4 isomerase (3β-HSD), 5α (β)-reductase, cytochrome P450175α, lyase, and 17β-HSD (17β-HSD), thus showing that the brain synthesizes a variety of neurosteroids from cholesterol, beginning with P5 and P5 organosulfates [1–5].

The cholesterol side-chain cleavage enzyme (P450scc) that synthesizes P5 from cholesterol, the basis of sex hormones, also exists in the retina, a central nerve, and may be involved in the production of neurosteroids [4]. E2 also has a neuroprotective effect in ailments such as Alzheimer’s disease and stroke [9,10], and there are also reports in the ophthalmological field that E2 plays a neuroprotective role when RGCs decline due to crush of the optic nerve [7,16,17]. In a study by Ishikawa et al [18], they cultivated separated optic cups of rats under high pressure and proved that the neurosteroid allo-P5 protected RGC axons from pressure-related injuries, and it has also been reported that neurosteroids may produce neuroprotective effects in cases of glaucoma.

The majority of E2 and T in the serum are thought to bind with sex hormone binding proteins and not pass through the blood-brain barrier or blood-retinal barrier [19]. Free E2 and T, as well as portions of sex hormones bound to albumin, move to the cerebrospinal fluid, but the rates of such movements are thought to be low. Since this present study focused on investigating diseases targeted for vitreous surgery, E2 and T concentrations in a normal vitreous body were not measured. In diabetic retinopathy, in which the blood-ocular barrier generally fails, it has been considered that free E2 and T in serum might possibly migrate into the vitreous body. However, in this study, despite the high levels of serum E2 and T in a male diabetic retinopathy patient, E2 and T concentrations in the vitreous in the other 3 diseases were low, so irrespective of blood-ocular barrier failure, we theorize that little migration from serum into the vitreous body occurred. Despite the fact that practically no transfer was found of T in the serum to the vitreous body in both the males and females in this study, our findings showed high concentrations of E2 in the vitreous body of the females, which has a similar structure to T, suggesting the possibility that E2 is synthesized locally in the eye.

While many of the females in this study were in the stages of menopause where ovary function is in decline, the reason for the males having a significantly higher concentration of E2 in the serum than the females is presumably that a high concentration of T secreted by the testicles is present in the blood of males, which is converted into E2 through the effects of aromatase present in cells such as adipose cells and fibroblast cells of the skin.

To elucidate the effects of neurosteroids in different kinds of conditions, it is vital to uncover the synthetic pathways of neurosteroids. Baulieu et al [1] reported that neurosteroids are synthesized by glial cells such as oligodendrocytes and astrocytes in the brain. Peterson et al [20] reported that E2 is produced by radial glia, etc., in the brain and that trauma to the brain causes reactive astrocytes to produce E2 and trigger neurogenesis, and similar findings have been reported in other studies [17,21]. Androstenedione and T are converted to E2 by the effects of aromatase in the synthesis of sex hormones, and when injuries occur in the brain, reactive astrocytes are said to manifest aromatase and contribute to the production of E2 [22,23]. The manifestation of aromatase is thought to rise not only during traumatic injury to the brain [23,24], but also with ischemia [25] and inflammation [26], and the pathology of the vitreoretinal diseases examined in this study (retinal disorders caused by MH, RD, retinal inflammation caused by DR and ERM, ischemia caused by DR, etc.) suggest the possibility that E2 is produced by an increased presence of aromatase through reactive astrocytes. It is believed that Müller cells, a major retinal glial cell, undergo reactive gliosis in the presence of such retinal diseases [27,28]. Therefore, not only astrocytes, but also Müller cells, may play an important role for producing E2 and involve the pathology of the vitreoretinal diseases.

Studies have reported that brain infarctions do not spread in females and produce few disabilities [29,30], suggesting the possibility that the production of aromatase is more elevated in the reactive astrocytes of females. There are further reports that aromatase is present in greater quantities in the brains of female mice [31], indicating that females perhaps have superior sexual dimorphism in astrocyte reaction toward different kinds of injury [32,33]. Sexual dimorphism is a polymorphic phenomenon in biology referring to a phenomenon wherein individual characteristics differ depending on gender. The quantity of aromatase produced in the brain and retina may also differ between males and females. If glial cells in the brain and retina, such as astrocytes, are taken to have the same characteristics, it could possibly serve as evidence explaining the higher concentrations of E2 in the vitreous body of all female retinas. However, it has also been reported that aromatase appears in the retinal pigment epithelium and retinal neuron [5,6], and further studies are needed to elucidate the relationship between vitreoretinal diseases and the production of aromatase in other cells aside from just glial cells, such as astrocytes.

We previously reported that E2 exists in high concentrations in the vitreous bodies of MH patients, however, we found no statistically significant differences in E2 concentrations between patients of MH and other diseases in this present study [34]. Although the reason for this is uncertain, our previous report used radioisotopes to perform measurements whereas the ECLIA method was utilized in this study, and perhaps the difference in measurement methods had an effect. Another possible reason is that the control group in our previous study included 3 cases of age-related macular degeneration. The E2 concentrations of those 3 cases were relatively low. However, there have also been reports that suggest the possibility that practically no E2 is detected in vitreous bodies unaffected by retinal lesions such as ERM, MH, DR, and RD [35], though interpretation is difficult due to no data being given on E2 concentrations in normal vitreous bodies. The presence of large amounts of fibrous astrocytes in the tissues of the ERM collected during MH surgeries has been reported [36], and these astrocytes may be similar to the reactive astrocytes that appear and produce aromatase when an injury is sustained to the brain. Accordingly, if E2 increases in the vitreous body of MH patients, it is conceivable that it is activated as a healing response to the injury and is possibly being produced by the retina’s astrocytes.

In conclusion, the findings of this study show higher E2 concentrations in the vitreous body than in the serum of female patients with different types of vitreoretinal disease. The underlying cause may be an elevated production of E2 from increased production of aromatase activated by reactive astrocytes as a result of the damage caused by each disease. Further multi-faceted studies are needed to elucidate the production of aromatase in the eye and gender differences, as well as to identify cells that produce E2 and the significance of E2 in individual diseases. Analyzing the dynamics and function of other neurosteroids in the retina, in addition to E2, might also lead to the future development of new treatments for vitreoretinal diseases.
